# Natural polyphenolic coffee extract administration relieves chronic nociplastic pain in a reserpine‐induced fibromyalgia‐like female mouse model

**DOI:** 10.1002/brb3.3386

**Published:** 2024-01-17

**Authors:** Rubén Toledano‐Martos, Anna Bagó‐Mas, Meritxell Deulofeu, Judit Homs, Núria Fiol, Enrique Verdú, Pere Boadas‐Vaello

**Affiliations:** ^1^ Research Group of Clinical Anatomy, Embryology and Neuroscience (NEOMA), Department of Medical Sciences University of Girona Girona Catalonia Spain; ^2^ University School of Health and Sport (EUSES), University of Girona Girona Catalonia Spain; ^3^ Department of Chemical Engineering, Agriculture and Food Technology, Polytechnic School University of Girona Girona Catalonia Spain

**Keywords:** nociplastic pain, polyphenols, reserpine‐induced myalgia

## Abstract

**Introduction:**

Nociplastic pain involves reflexive and nonreflexive pain responses and it is a core symptom of fibromyalgia (FM). The increasing prevalence of this health condition and the low rates of patients’ quality of life, combined with the lack of suitable pharmacologic treatments, evidence the demand to research new alternatives. Polyphenols may be potential therapeutic candidates as they have been reported to exert pathological pain modulation in preclinical models. In that context, this work was aimed to study the antinociceptive effects of a polyphenolic extract obtained from decaffeinated ground roasted coffee, in the RIM6 FM‐like mouse model.

**Methods:**

To this end, RIM6 adult ICR‐CD1 female mice were administered daily once a week with either 10 or 15 mg/kg of extract, and reflexive pain responses were evaluated for up to 3 weeks. At the end, the depressive‐like behavior was assessed as a nonreflexive pain response, and spinal cord and serum samples were collected for immunohistochemical and toxicological analyses.

**Results:**

These findings showed that the repeated administration of the coffee polyphenolic extract (CE) modulated reflexive pain responses, depressive‐like behavior, and spinal cord gliosis in a dose‐dependent manner, without signs of systemic toxicity.

**Conclusion:**

Thus, the CE may be a potential pharmacological treatment suitable to relieve nociplastic pain responses characteristic of FM.

## Graphical abstract

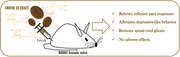

## 1 INTRODUCTION

Fibromyalgia (FM) is a chronic health condition mainly characterized by widespread pain (Coskun Benlidayi, 2019), usually accompanied by mood disorders like depression (Arnold et al., 2019; Coskun Benlidayi, 2019). As chronic widespread pain present in FM patients cannot be defined as nociceptive or neuropathic pain due to the lack of either neuronal or nonneuronal evident tissue damage as pain inducer, it is called nociplastic pain (Berwick et al., 2022; Fitzcharles et al., 2021; Kosek et al., 2021). That is, a pain that arises from altered nociception despite no clear evidence of actual or threatened tissue damage causing the activation of peripheral nociceptors or evidence for disease or lesion of the somatosensory system causing the pain (Treede et al., 2019). The underlying mechanisms of FM remain unknown, but recent studies suggest that neuroinflammation processes may play a pivotal role in FM pathogenesis (Correa‐Rodríguez et al., 2020; Coskun Benlidayi, 2019), in which microglia cells may be involved by releasing pain‐inducing proinflammatory factors (Coskun Benlidayi, 2019; Su et al., 2022). Despite the increasing knowledge about potential mechanisms, no suitable pharmacological strategies have been developed yet for FM treatment (Coskun Benlidayi, 2019). Considering the potential role of neuroinflammation in FM, further research in pharmacotherapy targeting the neuroinflammation processes is warranted.

In this context, a growing body of evidence suggests that polyphenols exert neuroinflammation modulatory effects in the nervous system leading to pathological pain responses decrease (Basu et al., 2021; Boadas‐Vaello et al., 2017; Guerrero‐Solano et al., 2020; Miguel et al., 2021; Szymaszkiewicz et al., 2022), and they would therefore be highlighted candidates for nociplastic pain alleviation. These findings together with recent evidence from refractory pathological pain studies suggest that single drug administration would be not effective enough (Pirvulescu et al., 2022), a suitable strategy may be the administration of a mixture of different polyphenols. In this regard, it has been shown that the administration of decaffeinated coffee polyphenolic extract (CE) in a mice model of spinal cord injury significantly attenuates the development of central neuropathic pain by modulating neuroinflammation processes (Bagó‐Mas et al., 2022). This coffee extract was well‐characterized by high‐performance liquid chromatography high‐resolution mass spectrometry (HPLC‐HRMS), and it consisted of chlorogenic acid, neochlorogenic acid, and cryptochlorogenic acid as major polyphenols (Bagó‐Mas et al., 2022). These polyphenols have been shown to exert pain‐modulating effects (Soler‐Martínez et al., 2022), but none of them at the same degree as the extract when administrated separately at the concentration in which they are found in the extract (Soler‐Martínez et al., 2022). Despite these results, the potential antinociceptive effects of this extract on nociplastic pain responses have not been evidenced yet.

In light of these findings, the present work aimed to determine whether the decaffeinated CE may exert antinociceptive effects in a FM‐like female model (RIM6 model) that develops chronic nociplastic pain and central nervous system gliosis (Álvarez‐Pérez, Bagó‐Mas, et al., 2022; Álvarez‐Pérez, Deulofeu, et al., 2022). To this end, RIM6 adult ICR‐CD1 female mice received 1‐week repeated administration of either 10 or 15 mg/kg of coffee extract, and reflexive and nonreflexive pain responses were assessed within 3 weeks of the experimental period. At the end, spinal cord samples were collected to determine whether RIM6‐gliosis related to nociplastic pain development is modulated by the treatment. In addition, serum levels of hepatotoxicity and nephrotoxicity biomarkers were analyzed to assess the pharmacological safety of the treatment.

## 2 MATERIALS AND METHODS

### 2.1 Animals and ethical considerations

Adult ICR‐CD1 mice (*n* = 34; 25–30 g) female mice were purchased in Janvier. Animals were housed in groups of 4–5 in standard Marcolon cages (28 × 28 × 15 cm^3^) under 12:12‐h light‐dark cycle, at 22 ± 2°C, and fed ad libitum with a standard diet of mouse pellets (TEKLAD 2014, Harlan Interfauna Ibérica).

All experimental procedures and animal husbandry were conducted following the ARRIVE 2.0 guidelines and performed according to the ethical principles of the IASP for the evaluation of pain in conscious animals (Zimmermann, 1983), and the European Parliament and the Council Directive of September 22, 2010 (2010/63/EU), along with the approval Ethical Committee on Animal Experimentation (CEEA) of the University of Barcelona and the Department of Agriculture, Livestock, Fisheries, Food and Natural Environment of the Generalitat de Catalunya, Government of Catalonia (DAAM number 9918). All experiments were carried out at the animal experimentation unit in the Bellvitge campus of the University of Barcelona, Catalonia.

### 2.2 Induction fibromyalgia‐like condition and experimental design

The reserpine‐induced myalgia RIM6‐mice mouse model of FM‐like condition was used in this study (Álvarez‐Pérez, Bagó‐Mas, et al., 2022). Reserpine (Sigma‐Aldrich) dissolved in acetic acid and diluted to a final concentration of 0.5% acetic acid with saline solution was administered subcutaneously (0.25 mg/kg) on days 0, 1, 2, 9, 16, and 23. At the same time‐points, CNT6 control animals received subcutaneous administration of reserpine dilution vehicle (saline solution).

Subsequently, during 1 week (week 4–5) starting from the fifth day after the last administration of reserpine (induction phase), RIM6 animals were administered once a day (i.p.) with either saline solution (RIM6‐Saline; *n* = 10), 10 mg/kg (RIM6‐CE10; *n* = 8), or 15 mg/kg (RIM6‐CE15; *n* = 8) of well‐quantified and HPLC‐characterized coffee extract (Bagó‐Mas et al., 2022), which are doses that have been shown to exert significant attenuative effects on neuropathic pain development in spinal cord injured‐mice (Bagó‐Mas et al., 2022; Soler‐Martínez et al., 2022). The coffee extract was obtained from commercial decaffeinated ground roasted coffee, a blend of Robusta natural (pure *Coffea canephora*) and Arabica natural (pure *Coffea arabica*) at a roast point of 7 over 10 (dark medium). The total amount of polyphenols was determined using the Folin–Ciocalteu assay, resulting in 2456 mg GAE/L. Fifteen control animals were also daily administered with saline solution (CNT6).

Reflexive pain responses (thermal hyperalgesia and mechanical allodynia) were weekly evaluated during the induction phase (weeks 0–4) and during the 3 weeks after treatment (post‐administration phase; weeks 5–7). Moreover, at the end of the experimental period, depression‐like behavior was also evaluated as a nonreflexive pain response. The animals were then sacrificed to obtain the spinal cord and serum samples for subsequent immunohistochemical and toxicological studies.

### 2.3 Reflexive and nonreflexive pain responses assessments

The reflexive pain responses thermal hyperalgesia and mechanical allodynia were evaluated as previously described (Álvarez‐Pérez, Bagó‐Mas, et al., 2022; Álvarez‐Pérez, Deulofeu, et al., 2022; Bagó‐Mas et al., 2022; Soler‐Martínez et al., 2022). Thermal hyperalgesia was assessed using an algesimeter (37370; Ugo Basile), with a radiant heat source of 100 W. A cut‐off of 25 s hind paw exposition was established to prevent skin damage. Von Frey monofilaments (bending force range, 0.04–2 g) were used for mechanical allodynia assessment. Overall, 50% withdrawal thresholds of the hind paws to filaments stimulation were measured following the up–down paradigm, and the final outcomes were obtained after Dixon formula application (Dixon, 1980). Both hind paws were evaluated for reflexive pain responses assessments.

Regarding nonreflexive, the forced swimming test was performed following procedures explained elsewhere (Álvarez‐Pérez, Bagó‐Mas, et al., 2022; Álvarez‐Pérez, Deulofeu, et al., 2022). Briefly, it consisted of forcing the mice to swim in 40 cm high × 15 cm diameter glass containers (containing 30 cm of water at 25 ± 1°C). Its behavior was recorded for 6 min using a videocam (Sony HDR‐CX190). The recordings were analyzed to determine the mobility and immobility time of the animals. The immobility time was determined when no additional activity was observed other than the movements required to keep the mouse head above the water. A depressive‐like behavior animal exhibits more extended immobility (Álvarez‐Pérez, Bagó‐Mas, et al., 2022; Álvarez‐Pérez, Deulofeu, et al., 2022; Porsolt et al., 1977).

### 2.4 Immunohistochemical analysis

At the end of the experimental period, the animals were deeply anesthetized with sodium pentobarbital (90 mg/kg; i.p.) and the spinal cord segment distal to T10 was collected to determine both microgliosis and astrogliosis. The immunohistochemical analyses were performed following the processes previously described (Álvarez‐Pérez, Bagó‐Mas, et al., 2022; Álvarez‐Pérez, Deulofeu, et al., 2022; Bagó‐Mas et al., 2022; Soler‐Martínez et al., 2022). The rabbit anti‐adaptor molecule 1 ionized calcium binding agent (Iba1; 1:200; 019‐19‐741; WAKO) and rabbit anti‐GFAP (Glial fibrillary acidic protein; 1:200, ab7260, Abcam) were used for immunostaining microglia and astroglia cells, respectively, in 60‐μm spinal cord sections. The donkey anti‐rabbit secondary antibody conjugated with cyanine 3.18 (1:200, Cy3, Jackson ImmunoResearch) was used to visualize the cells by epifluorescence. A minimum of five dorsal horn images (×200) obtained by a digital camera (FMVU‐13S2CCS, Point Gray Research) coupled to a microscope equipped with epifluorescence (Leica DMR‐XA; Leica Microsystems) were evaluated using NIH Image software (ImageJ; version 1.37; National Institutes of Health). The number of reactive and nonreactive microglial cells was determined according to their morphology (Kettenmann et al., 2011). The percentage of reactive microglia cells and the GFAP immunoreactivity area were used as an index of microgliosis and astrogliosis degree, respectively (Álvarez‐Pérez, Bagó‐Mas, et al., 2022; Álvarez‐Pérez, Deulofeu, et al., 2022; Bagó‐Mas et al., 2022; Soler‐Martínez et al., 2022).

### 2.5 Biochemical analysis of hepatotoxicity and nephrotoxicity

Serum samples were analyzed to rule out the potential hepatotoxicity and nephrotoxicity processes associated with the polyphenolic treatments. The levels of alanine aminotransferase (ALT/GPT; 11533, BioSystems), aspartate aminotransferase (AST/GOT; 11531, BioSystems), and urea‐BUN (11536, BioSystems) were analyzed in the serum samples by using commercial assay kits (Bagó‐Mas et al., 2022; Soler‐Martínez et al., 2022).

### 2.6 Statistical analysis

All functional and histological analyses were performed in a blinded manner using a numeric code for each mouse. The normal distribution of the data was analyzed with a Kolmogorov–Smirnov test before further statistical analyses. When data followed a normal distribution, the repeated measures Multivariate analysis of variance (MANOVA) (Wilks’ criterion) and/or analysis of variance (ANOVA) followed by Duncan's test were performed. When data did not follow a normal distribution, outcomes were analyzed using a Friedman statistic test for nonparametric repeated measures and/or Kruskal–Wallis followed by Bonferroni's test. Finally, a principal component analysis (PCA) was carried out using functional and histological variables assessed in the previous statistical tests. Data were illustrated in figures as mean ± SEM or median ± Interquartile range (IQR). The significance level α was set at 0.05, using the IBM SPSS 25.0 statistical package for Windows (IBM Corp. Released 2017).

## 3 RESULTS

### 3.1 Coffee extract repeated administration results in significant reflexive pain responses relief

**FIGURE 1:**
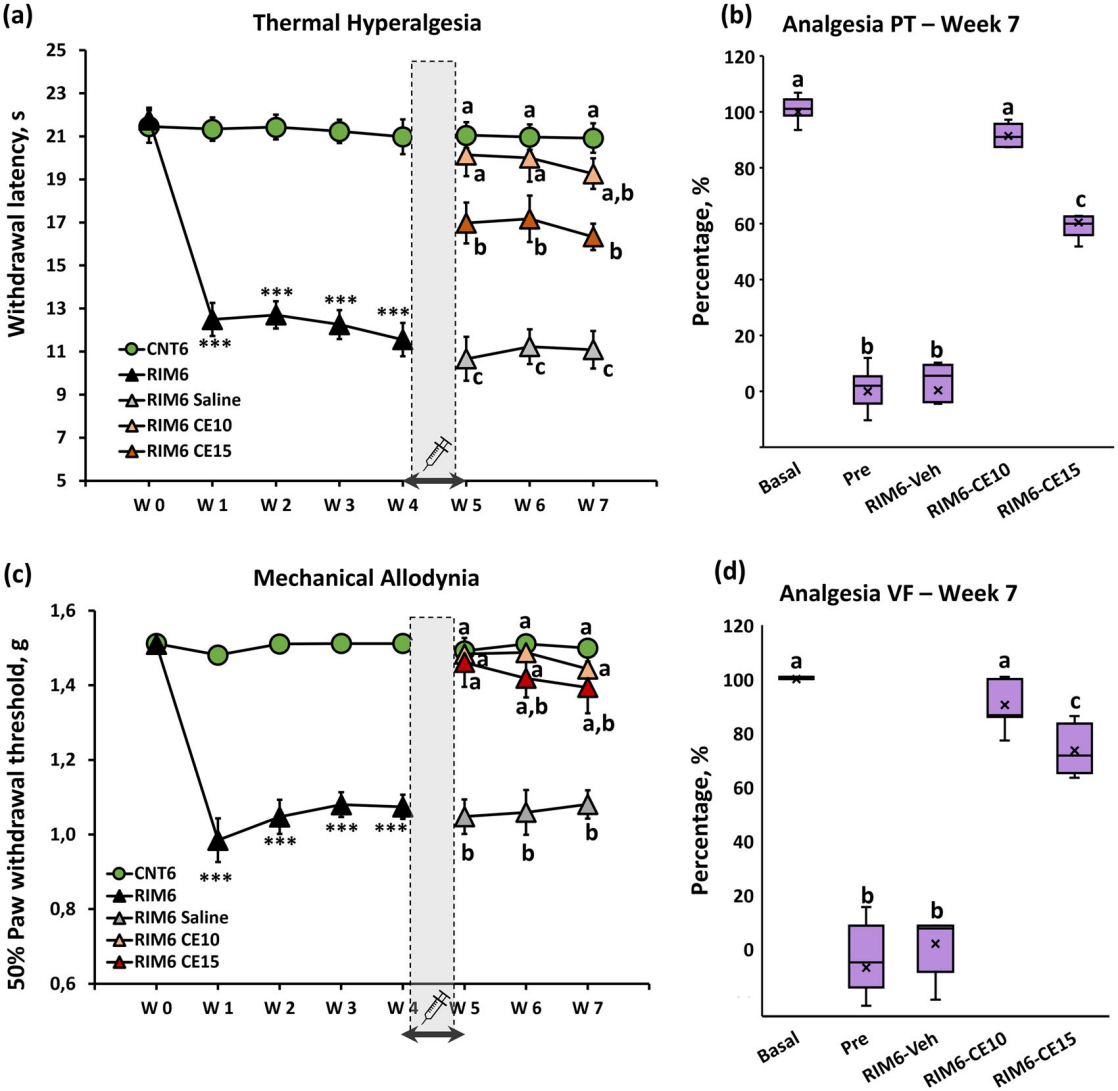
Weekly results of (a) thermal hyperalgesia (c) mechanical allodynia during the reserpine‐induction period (CNT6 and RIM6 from W0 to W4) and the posttreatment period (CNT6, RIM6‐Saline, RIM6 CE10, and RIM6 CE15 from W4 to W7); and percentage of analgesia 3 weeks after administration in terms of (b) thermal hyperalgesia and (d) mechanical allodynia. The shaded bar and the syringe indicate the administration week (W4–W5). Each point and vertical line represent the mean of reflexive responses ± SEM. Data of analgesia are expressed as median ± IQR, and the mean is also shown as a “×” ***p < .0001 versus CNT6. (a and b) Groups not sharing a letter after treatment are significantly different, *p* < .05, by corresponding post hoc test.

Regarding thermal hyperalgesia, the Kolmogorov–Smirnov normality test revealed normal distribution of data in all the time‐points of functional assessment (*p*s > .05). During the induction experimental phase, the MANOVA analysis indicated significant effects on week (*p* < .001) and treatment (*p* < .001) factors and significant interaction for week × treatment (*p* < .001). On further ANOVA analysis, whereas significant group differences were found on weeks 1–4 (all *p*s < .001), no significant differences were found before induction protocol (week 0, *p* = .269). These results indicate that RIM6 mice showed significant decrease in paw withdrawal latency to thermal stimulation in comparison to CNT6 animals during the induction phase (Figure 1a). As for the post‐administration phase, the MANOVA analysis indicated significant effects on week (*p* = .013) and treatment (*p* < .001) factors and significant interaction for week × treatment (*p* = .004). On further ANOVA analysis, significant group differences were found on weeks 5–7 (all *p*s < .001) (Figure 1a). Concretely, the subsequent post hoc analysis revealed a significant (*p* < .05) increase of paw withdrawal latency to thermal stimulation in both RIM6‐CE10 and RIM6‐CE15 mice in comparison with RIM6‐Saline during the whole post‐administration phase. Moreover, a lack of significance was observed during this period between RIM6‐CE10 and CNT6 groups indicating a significant complete alleviation of thermal hyperalgesia of RIM6 animals treated with 10 mg/kg of coffee extract. In terms of percentage of analgesia, both doses exerted significant effect when compared with pre‐administration and vehicle administration (all *p*s < .015), being significant higher the dose of 10 mg/kg (91.41%) than 15 mg/kg (60.43%) (Figure 1b).

As for mechanical allodynia, in contrast to thermal hyperalgesia, the Kolmogorov–Smirnov normality test revealed that mechanical allodynia data did not follow a normal distribution at any time point of functional assessment (*p*s < .001). Thus, nonparametric tests have been used to analyze Von Frey data. The Friedman test revealed that the distribution of the mechanical allodynia data significantly varied during the experimental period (*p* = .014). On further post hoc test analysis, significant group differences were found during induction experimental phase, on weeks 2–5 (all *p*s < .036) (Figure 1c) indicating that RIM6 mice developed mechanical allodynia in comparison with CNT6. During the post‐administration experimental phase, the Kruskal–Wallis test indicated differences between groups at weeks 5, 6, and 7 (all *p*s < .001). Concretely, subsequent post hoc analysis revealed a significant increase in paw withdrawal of RIM6‐CE10 when compared with RIM6‐Saline during the whole phase (all *p*s < .06). RIM6‐CE15 also showed a significant decrease in comparison with RIM6‐Saline RIM6‐CE15 at week 5 (*p* = .06) but a lack of significance was observed between RIM6‐CE15 with either RIM6‐Saline or RIM‐CE10 at weeks 6 and 7 (all *p*s > .05) (Figure 1c). Like thermal hyperalgesia, both doses exerted significant percentage of analgesia when compared with pre‐administration and vehicle administration (all *p*s < .012), although higher effects were revealed after the dose of 10 mg/kg (90.43%) than 15 mg/kg (73.52%) (Figure 1d).

### 3.2 Coffee extract repeated administration relieves depression‐like behavior, the main nonreflexive pain response in fibromyalgia‐like RIM6 model

**FIGURE 2:**
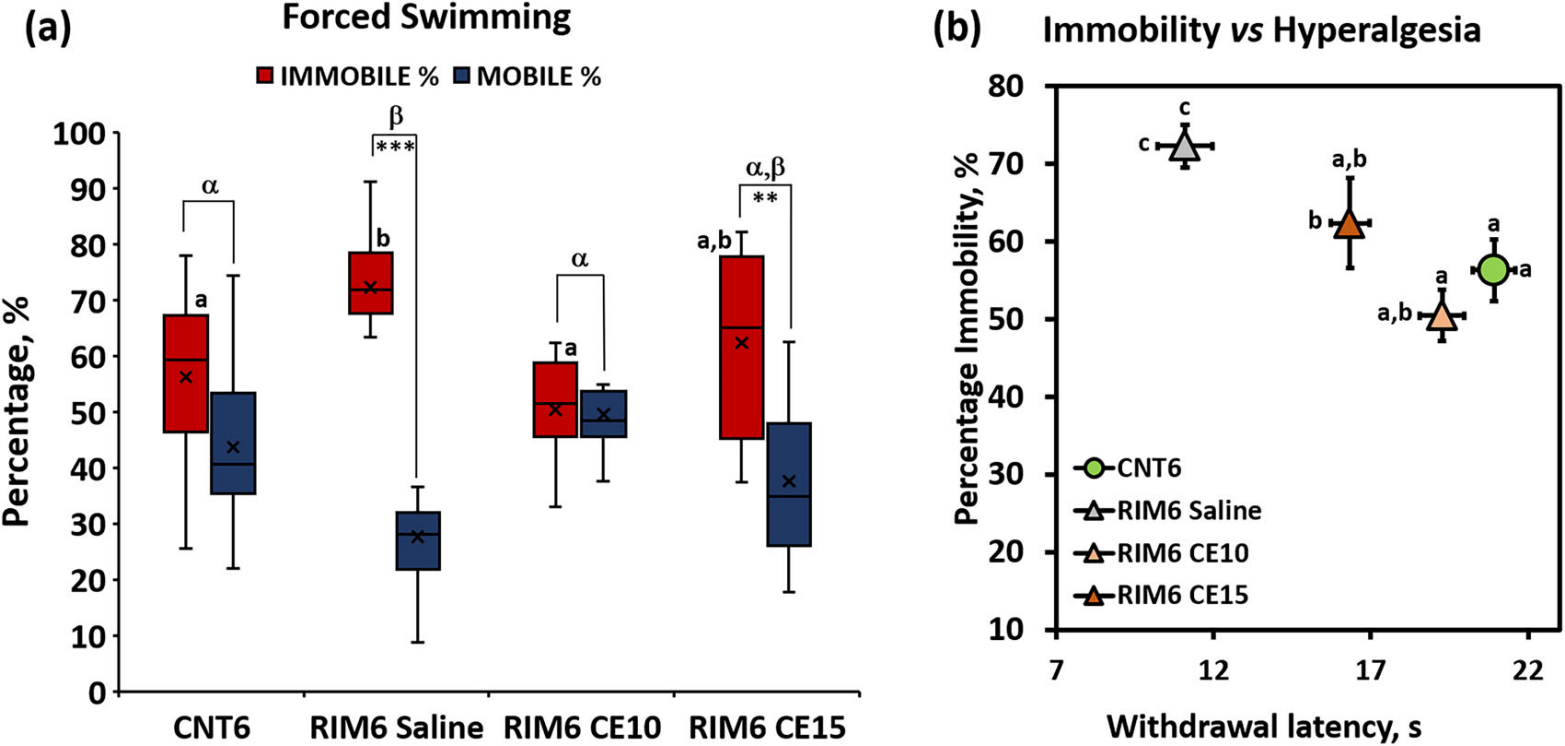
(a) Percentage of immobility and mobility time in the forced swimming test of CNT6, RIM6‐Saline, RIM6 CE10, and RIM6 CE15 3 weeks after the first treatment administration. Data are expressed as median ± IQR, and the mean is also shown as a “×.” (a and b) Groups not sharing a letter showed significant differences in %Immobility time, *p* < .05; a–b: Groups not sharing a letter showed significant differences in %Immobility‐%Mobility time *p* < .05; intragroups significant differences: ***p < .0001 %immobility versus %mobility, *p < .05 %immobility versus %mobility. (b) Relationship between reflexive and nonreflexive pain responses of CNT6, RIM6‐Saline, RIM6 CE10, and RIM6 CE15 3 weeks after the first treatment administration. Data are expressed as the mean ± SEM. (a–c) Groups not sharing a letter are significantly different, *p* < .05; letters above the symbols correspond to the percentage of immobility in the forced swimming test, and letters to the left of the symbols refer to thermal hyperalgesia data.

The Kolmogorov–Smirnov normality test revealed normal distribution of forced swimming data (*p*s > .05). The ANOVA analyses showed significant group differences for both “%Immobility time” and “%Immobility‐%mobility” variables (all *p*s < .002) (Figure 2a). The subsequent post hoc analysis revealed no significant differences between CNT6 and either RIM6‐CE10 or RIM6‐CE15 (*p* > .05), indicating similar immobility in these groups. In contrast, the RIM6‐Saline group showed a significant increase in immobility when compared with either CNT6 or RIM6‐CE10 (*p* < .05). Finally, RIM6‐CE15 mice immobility could not be significantly distinguished from either RIM6‐Saline or RIM6‐CE10 (Figure 2a). In parallel, although intragroups ANOVA analysis of %Mobility and %Immobility showed a lack of significant differences in both CNT6 (*p* = .052) and RIM6‐CE10 (*p* = .846), a significant increase in immobility time was observed in both RIM6‐Saline and RIM6‐CE15 groups.

### 3.3 Coffee extract repeated administration significantly reverses spinal cord gliosis

**FIGURE 3:**
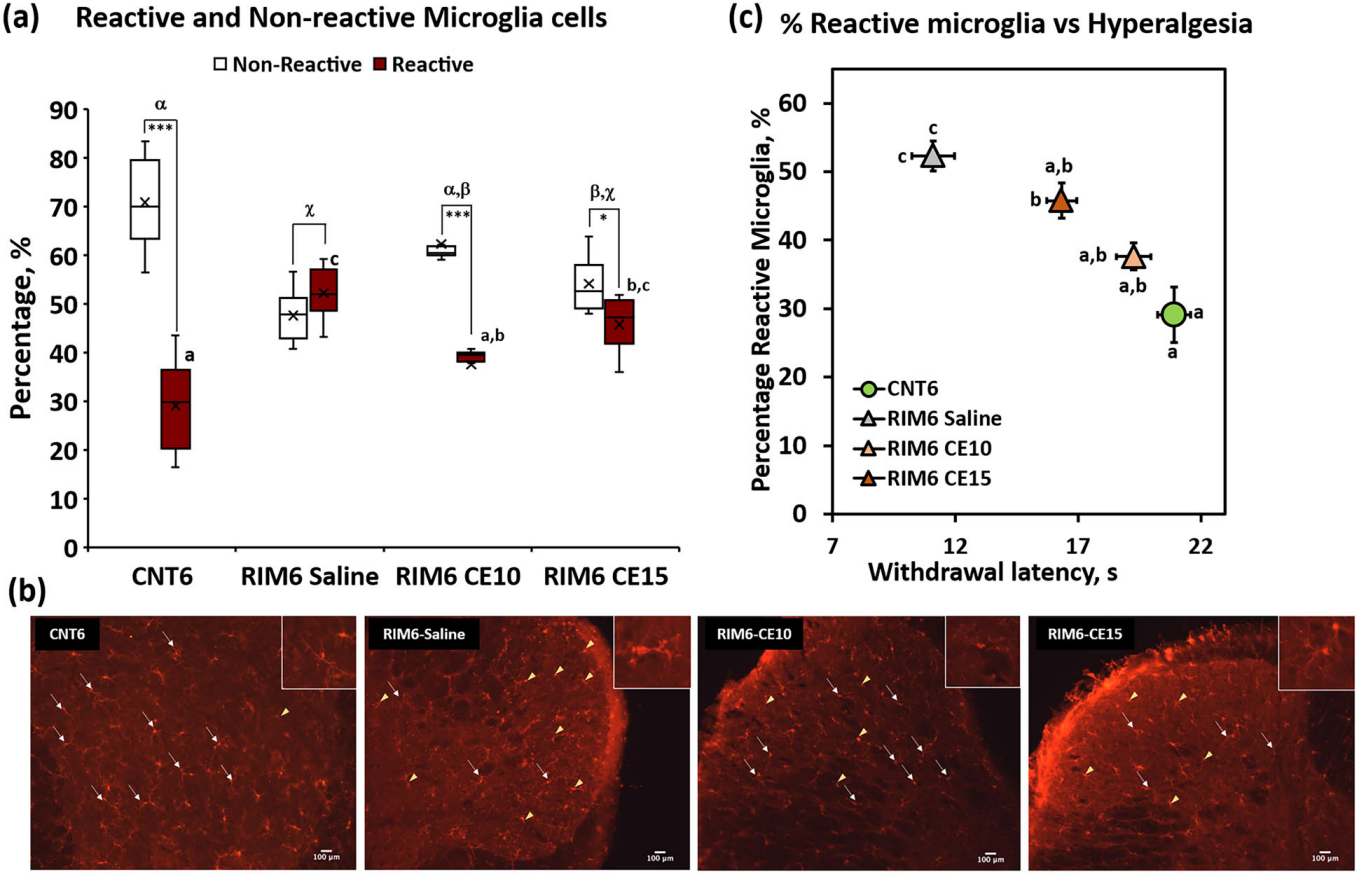
(a) Percentage of reactive and nonreactive microglia cells in the spinal cord DH of CNT6, RIM6‐Saline, RIM6 CE10, and RIM6 CE15 3 weeks after the first treatment administration, performed 5 days after the last administration of reserpine. Data are expressed as median ± IQR, and the mean is also shown as an “×.” (a–c) Groups not sharing a letter showed significant differences in %reactive cells, *p* < .05; α–χ: Groups not sharing a letter showed significant differences in %nonreactive‐%reactive, *p* < .05; intragroups significant differences: ***p < .0001 %nonreactive versus %reactive, *p < .05 %Nonreactive versus %reactive. (b) Representative medullary sections of the DH. The images show the reactivity of the microglia cells by marking Iba‐1. The triangle indicates the reactive cells, whereas the arrow indicates the nonreactive cells. Scale bar 100 μm. (c) Relationship between thermal hyperalgesia and percentage of reactive microglia cells in the dorsal horn of CNT6, RIM6‐Saline, RIM6 CE10, and RIM6 CE15 3 weeks after the first treatment administration, performed 5 days after the last administration of reserpine. Data are expressed as the mean ± SEM. (a–c) Groups not sharing a letter are significantly different, *p* < .05; letters above the symbols correspond to the percentage of reactive cells, and letters next to the symbols (right or left) refer to thermal hyperalgesia data.

Regarding the microgliosis, the Kolmogorov–Smirnov normality test revealed normal distribution of the data corresponding to the percentage of reactive and nonreactive microglia cells in the spinal cord (all *p*s > .05). The ANOVA analysis of the immunohistochemistry images of the dorsal horn of the spinal cord revealed significant group differences (all *p*s < .001). Concretely, although the subsequent post hoc analysis showed a significant (*p* < .005) increase in the percentage of reactive microglia cells in RIM6‐Saline mice when compared with CNT6 (Figure 3a), such percentage was significantly decreased (*p* < .005) in RIM6‐C10 group in comparison to RIM6‐Saline, reaching similar levels of CNT6. Finally, the percentage of reactive microglia cells in RIM6‐C15 could not be significantly differentiated from either RIM6‐Saline or RiM6‐CE10. Representative images from the sections of the spinal cord dorsal horn visualized in the microscope are shown in Figure 3b. As for the intragroup analysis of the cell types percentages, no differences were observed in RIM6‐Saline (*p* = .168), whereas a significant higher percentage of nonreactive versus reactive cells (*p* < .0001) were observed in both CNT6 and RIM6‐CE10 (Figure 3a). Finally, despite the similarity between RIM6‐Saline and RIM6‐CE15, the latter showed a slight significant percentage of nonreactive when compared with reactive cells (*p* = .045) (Figure 3a).

**FIGURE 4:**
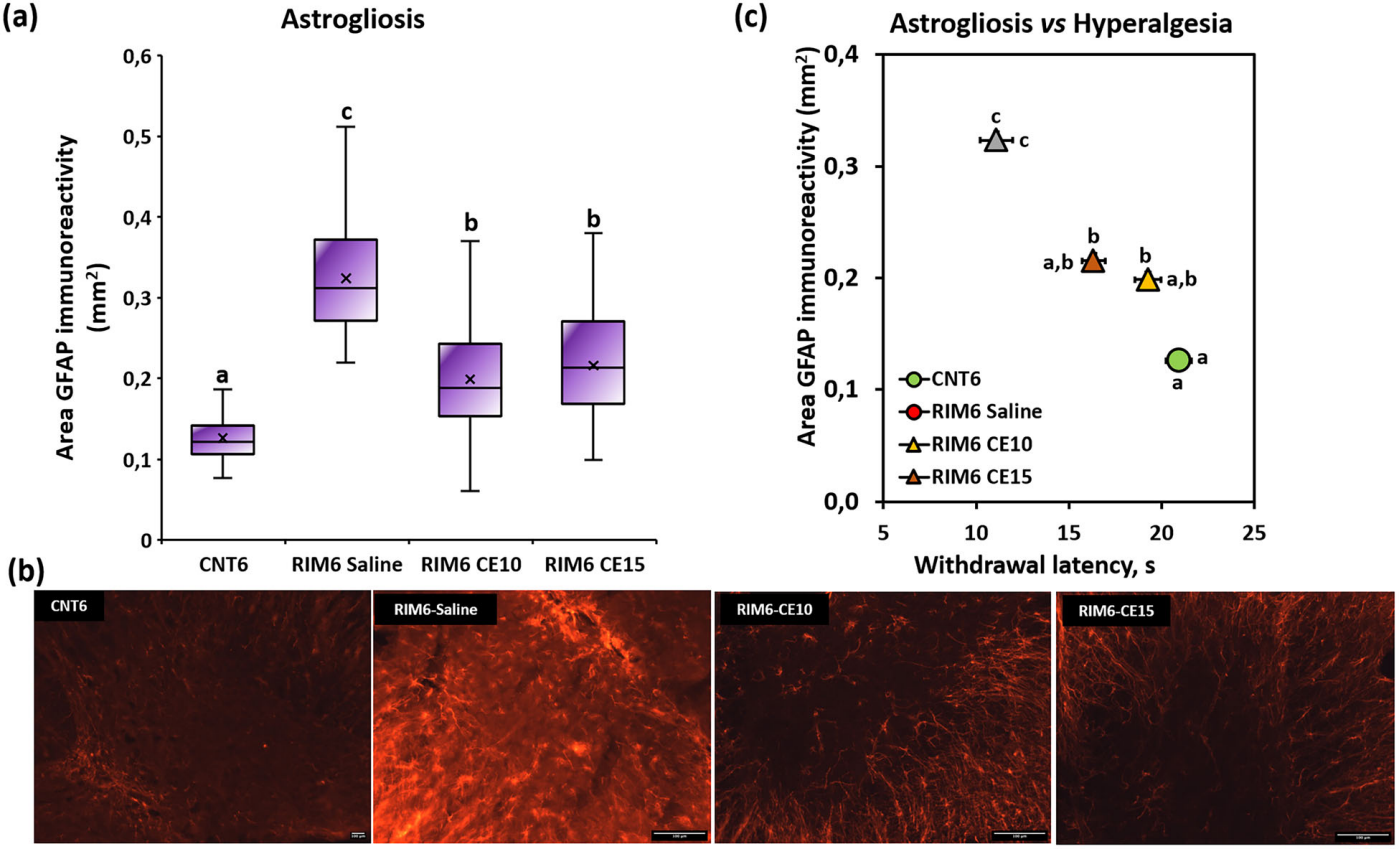
(a) Representative histological images of the spinal cord immunostained against glial fibrillary acidic protein (GFAP) of CNT6, RIM6‐Saline, RIM6 CE10, and RIM6 CE15 3 weeks after the first treatment administration, performed 5 days after the last administration of reserpine. Data are expressed as median ± IQR, and the mean is also shown as an “×.” (a–c) Groups not sharing a letter showed significant differences in GFAP immunoreactivity area, *p* < .05; (b) representative histological images of the spinal cord immunostained against GFAP of each experimental group. Scale bar 100 μm. (c) Relationship between thermal hyperalgesia and GFAP immunoreactivity area in the dorsal horn of CNT6, RIM6‐Saline, RIM6 CE10, and RIM6 CE15 3 weeks after the first treatment administration, performed 5 days after the last administration of reserpine. Data are expressed as the mean ± SEM. (a–c) Groups not sharing a letter are significantly different, *p* < .05; letters above the symbols correspond to the GFAP immunoreactivity area, and letters next to the symbols (right or left) refer to thermal hyperalgesia data.

Regarding spinal cord astrogliosis assessment, the Kolmogorov–Smirnov test revealed non‐normal distribution of the GFAP immunoreactivity area data (*p* < .0001) and the subsequent Kruskal–Wallis analysis showed significant group differences (all *p*s < .001). Specifically, further post hoc analyses indicated that the significant astrogliosis observed in the RIM6‐Saline mice, when compared with CNT6 (*p* < .0001) (Figure 4a), was significantly decreased in both RIM6‐C10 and RIM6‐C15 in comparison to RIM6‐Saline. No differences were observed between RIM6‐C10 and RIM6‐C15 experimental groups (*p* > .005) (Figure 4a).

### 3.4 The administration of coffee extract exerts antinociceptive effects without adverse effects in RIM6 mice

**FIGURE 5:**
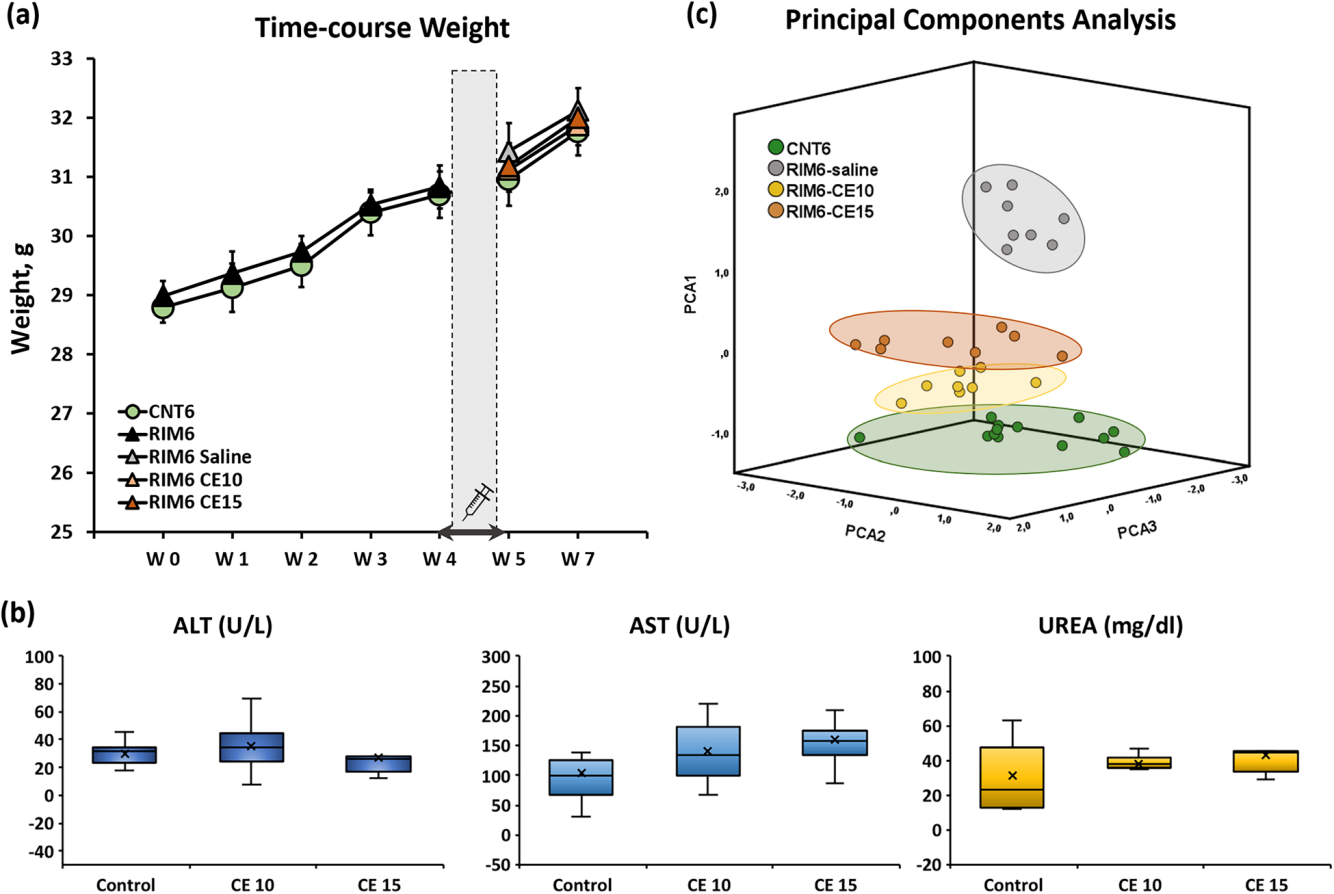
(a) Weekly results of animals weigh during the reserpine‐induction period (CNT6 and RIM6 from W0 to W4) and the posttreatment period (CNT6, RIM6‐Saline, RIM6 CE10, and RIM6 CE15 from W5 to W7). No significant changes in weight between groups were detected throughout the experimental period. Administration week (W4 to W5) is highlighted with coffee beans vector. (b) Biomarkers quantification of hepatotoxicity and nephrotoxicity, in the serum of controls and mice treated with CE10 or CE15 3 weeks after the first treatment administration. Data are expressed as median ± IQR, and the mean is also shown as an “×.” No differences between groups were shown in any of the parameters. (c) Principal component analysis, at the end of the experimental period. 3D score plot in which all animals are represented regarding the 3 principal components. Graph orientation: vertical = 351, horizontal = 131.

Following a protocol animal welfare supervision based on Morton D.B. and Griffiths P.H. guidelines (Morton & Griffiths, 1985), changes in coat and skin, vibrissae of the nose, nasal secretions, signs of autotomy of hindpaw and/or forepaw, or aggressiveness were not detected in mice after administering the reserpine (s.c.) or the CE (i.p.) at any time of the experimental period. Regarding the weight control, the Kolmogorov–Smirnov normality test revealed normal distribution of weight data throughout the experimental period (all *p*s > .058). In parallel, the MANOVA analysis indicated a lack of significant effects in the week (*p* = .084), treatment (*p* = .061), and week × treatment (*p* = .593) factors during the induction period. During the treatment period, the MANOVA analysis indicated significant effects in the week factor (*p* < .001) but a lack of significance of both treatment (*p* = .711) and interaction for week × treatment (*p* = .990) factors. These results indicate that reserpine administration did not exert any significant detrimental effects on the animals’ weight. Moreover, these results indicate also that there is a significant increase in weight during the treatment period, but this increase affected equally to all of experimental animals (Figure 5a). As for biochemical analysis of hepatotoxicity and nephrotoxicity, the Kolmogorov–Smirnov normality test revealed normal distribution of the ALT, AST, and UREA data (all *p*s > .05). The further ANOVA analysis of the biomarkers ALT, AST, and UREA of the mice serum revealed nonsignificant group differences (all *p*s > .05) (Figure 5b).

## 4 DISCUSSION

Thermal hyperalgesia and mechanical allodynia are known to be characteristic reflexive pain responses of pathological pain, including therefore the nociplastic pain (Fitzcharles et al., 2021) present in FM condition (Arnold et al., 2019). In this study we evaluated whether the CE may exert antinociceptive effects in the RIM6 preclinical model of FM‐like syndrome (Álvarez‐Pérez, Bagó‐Mas, et al., 2022; Álvarez‐Pérez, Deulofeu, et al., 2022), using the effective doses assessed in a model of central neuropathic pain (Bagó‐Mas et al., 2022; Soler‐Martínez et al., 2022). Our findings indicated that decaffeinated CE significantly relieves chronic nociplastic pain in RIM6 FM‐like model as significant increase of both paw withdrawal latency to thermal stimulus and paw withdrawal threshold to mechanical stimulus was observed in either RIM6‐CE10 or RIM6‐CE15 mice when compared with RIM6‐Saline. Indeed, the percentage of analgesia was even higher than observed after acute administration of pregabalin in RIM6 mice (Álvarez‐Pérez, Bagó‐Mas, et al., 2022), which is one of the FDA recommended drugs for FM (Ablin & Häuser, 2016). It is worth mentioning that polyphenols‐based treatments have been previously assessed on nociplastic pain models (Kaur et al., 2020; Peres Klein et al., 2016; Singh et al., 2021), but available data so far involve only short‐term effects. In contrast, the CE exerts long‐lasting significant reflexive pain responses relief.

Interestingly, the CE10 dose exerts better antinociceptive effects than CE15. These results are consistent with previous results shown in spinal cord‐injury neuropathic pain female mice model (Bagó‐Mas et al., 2022; Soler‐Martínez et al., 2022). Although the mechanisms by which CE would have antinociceptive effects are not yet fully elucidated, it is not unreasonable to suggest that CE15 did not exert better benefits likely because it exceeded the selectivity threshold of CE polyphenols for their primary targets, and off‐target effects may arise and exert counteracting or undesired effects. This controversial effect has been shown also in RIM6 mice after pregabalin administration (Álvarez‐Pérez, Bagó‐Mas, et al., 2022). Further pharmacokinetic experiments should be carried out once more information on the therapeutic targets of polyphenols becomes available.

On the other hand, it worth to note that chronic or pathological pain may also be related to the behavioral and persistent component of pain (Backonja & Stacey, 2004; Negus et al., 2006; Rice et al., 2008) and therefore, the evaluation of evoked‐reflexive pain responses may be not sufficient to make a comprehensive pain assessment. That is, pain is not just a reflex, but rather a perceptual experience with powerful emotional components, and these nonreflexive responses provide more comprehensive information about nociceptive input and its impact to the whole animal, more in line with the subjective description of real pain (Tappe‐Theodor & Kuner, 2014; Treede et al., 2019). In this context, besides the reflexive pain responses, in the present work it was also assessed whether the depression‐like behavior, one of the major nonreflexive pain responses shown in both FM‐patients (Arnold et al., 2008; Coskun Benlidayi, 2019), and RIM6 mice model (Álvarez‐Pérez, Bagó‐Mas, et al., 2022; Álvarez‐Pérez, Deulofeu, et al., 2022), would be reduced by CE repeated administration. Data were obtained by forced swimming test and revealed a significant decrease of immobility time after CE treatment in comparison to RIM6‐Saline, especially when CE10 was administered. Overall, these findings suggest that CE treatment administered during one week after nociplastic pain induction may modulate the depression‐like behavior significantly developed in RIM6‐Saline mice, with being CE10 the most effective dose. In addition, these results are consistent with reflexive pain responses data. That is, these nonreflexive outcomes may be associated with those obtained in the Hargreaves test indicating a relationship between thermal hyperalgesia and increased depressive‐like behavior in the RIM6‐Saline group (Figure 2b), being reverted by CE10 administration. Hence, CE10 treatment not only exerts significant alleviation of reflexive pain responses but may also revert the depression‐like behavior, the main nonreflexive pain response in the RIM6 FM‐like model.

Although no molecular analyses have been performed yet in supraspinal structures associated with affective nonreflexive pain responses in RIM6 model mice, some pieces of evidences suggest that increased neuroinflammatory‐related biomarkers are present in reserpined rodents, such as substance‐*P*, TNFα, and GluN2B‐NMDA‐receptor (Arora & Chopra, 2013; Singh et al., 2021). Considering the pivotal role of polyphenols in neuroinflammation modulation (Basu et al., 2021; Boadas‐Vaello et al., 2017; Guerrero‐Solano et al., 2020; Miguel et al., 2021; Szymaszkiewic et al., 2022), new experiments stating the downregulation of this kind of molecules should further be performed to confirm this hypothesis. On the other, distal release of inflammatory molecules, synthesized by damaged cells in the spinal cord after injury, has been proposed as a molecular mechanism that triggers microglial activation at distant spinal segments and supraspinal structures (Hulsebosch et al., 2009; Zhao et al., 2007). Considering that spinal cord microgliosis may play an essential role in the nociplastic pain development (Álvarez‐Pérez, Bagó‐Mas, et al., 2022; Álvarez‐Pérez, Deulofeu, et al., 2022), it can be suggested that secondary to this pathophysiological processes, inflammatory mediators may activate supraspinal neuroinflammation by gliosis activation that could explain the development of nonreflexive pain responses. Indeed, it has been suggested an increase of microgliosis in supraspinal structures in reserpined rats (D'Amico et al., 2021; Fusco et al., 2019) that would be a therapeutic target for FM‐like condition relief.

On the other hand, spinal cord gliosis has been suggested to play a crucial role in nociplastic pain responses development, as shown in the RIM6 mice (Álvarez‐Pérez, Bagó‐Mas, et al., 2022; Álvarez‐Pérez, Deulofeu, et al., 2022). To gain mechanistic insights, the modulation of glial cell activation was assessed after CE repeated treatment. Our findings indicated that CE repeated treatment exerts glia reactivity attenuation, as both micro‐ and astrogliosis were significantly decreased in either RIM6‐CE10 and RIM6‐CE15 experimental groups. In addition, it is worth mentioning that these results associate with those obtained in the Hargreaves test indicating a relationship between thermal hyperalgesia and increased reactive microglia cells and astrogliosis in the RIM6‐Saline group (Figures 3c and 4c), being modulated by CE treatment and consequently relieving thermal hyperalgesia. In fact, it has recently been described that CE may modulate spinal cord gliosis development after spinal cord injury (Bagó‐Mas et al., 2022; Soler‐Martínez et al., 2022). In this case, knowing that both micro‐ and astrogliosis are present in the spinal cord of RIM6 animals (Álvarez‐Pérez, Bagó‐Mas, et al., 2022), data are shown that CE may significantly attenuate spinal gliosis that is already developed at 4 weeks after reserpine inducing. In contrast to spinal cord injury in which gliosis is related to spinal contusion (Bagó‐Mas et al., 2022; Soler‐Martínez et al., 2022), in the RIM6 model it has been suggested to be associated with the lower release of descending fibers biogenic amines after reserpine administration (Álvarez‐Pérez, Bagó‐Mas, et al., 2022). Regarding the molecular mechanisms that may explain this beneficial effect, they are still poorly understood. It has been suggested that synergistic or summation effects of the major polyphenols present in the extract may contribute to modulate crucial pathways of central sensitization (Soler‐Martínez et al., 2022). Among them, the modulation of chemokine axes like CCL2/CCR2 and CX3CL1/CX3CR1 pathways may be highlighted as it is known that CE significantly prevents either CCR2 or CX3CR1 overexpression after spinal cord injury (Bagó‐Mas et al., 2022). Both CCR2 and CX3CR1 are upregulated in microglia, astrocytes, and neurons (Chen et al., 2020; Komiya et al., 2020; Rong et al., 2021), and their activation leads to inflammatory mediators’ release. However, the involvement of these axes’ modulation needs to be studied to better elucidate the CE mechanisms on nociplastic pain relief.

From a translational point of view, it is just as important to study the beneficial effects of CE as to ensure the absence of negative side effects. Thus, welfare, hepatotoxicity, nephrotoxicity, and weight control assessments were performed to determine whether CE treatments were safe. Results indicated no systemic toxicity associated with either CE10 or CE15 administration and neither hepatotoxic nor nephrotoxic effects. These findings are consistent with previous data showing no systemic toxicity after CE repeated treatment in other animal models (Bagó‐Mas et al., 2022; Soler‐Martínez et al., 2022). Indeed, CE was obtained using physiological serum as extracting agent (saline solution) instead of organic solvent that would be incompatible for administration to animals (Bagó‐Mas et al., 2022), making easier the potential translation into the clinics. Furthermore, considering the available evidence that not only the coffee extract but also other natural extracts with no adverse effects could be suitable for pathological pain treatment (Bagó‐Mas et al., 2022) or other inflammatory disorders (Atanasov et al., 2021; Schmidt et al., 2007), it is not unreasonable to hypothesize that the combination several natural polyphenolic extracts may be also a not available yet potential therapeutic strategy for patients suffering nociplastic pain conditions.

## 5 CONCLUSIONS

Altogether, data indicate that the decaffeinated CE intraperitoneally administered for 1‐week after reserpine‐induced nociplastic pain development in female mice results in significant relief of chronic reflexive (thermal hyperalgesia and mechanical allodynia) and nonreflexive (depression‐like behavior) pain responses up to 3 weeks after the first day of treatment. Moreover, the relationship between pain responses and central glia reactivity 3 weeks after treatment suggests that CE effects may result from nociplastic pain‐related microgliosis modulation. CE 10 mg/kg would be the most suitable dose since exerted the most significant beneficial effects during the weeks after treatment when compared with RIM6 animals treated with vehicle.

Interestingly, when functional and histological data were analyzed by PCA and represented on a 3D score plot, it could be observed that RIM6‐veh mice were grouped in a cluster which was clearly separated from the other animals (Figure 5c). On the other hand, although RIM6‐CE15 mice were grouped in a differentiated cluster, CNT6 and RIM6‐CE10 animal clusters were clearly overlapped. Hence, when the dimensionality of the whole data is reduced by means of generating principal component variables, this clearly supports the significant beneficial effects of CE on RIM6 FM‐like models, being the CE10 the most advantageous. In conclusion, data suggest that CE polyphenolic extract may be a potential pharmacological treatment suitable to relieve reflexive and nonreflexive pain responses, which are characteristic symptoms of FM syndrome.

## AUTHOR CONTRIBUTIONS

**Rubén Toledano‐Martos**: Methodology; validation; formal analysis; investigation; data curation; writing—review and editing; visualization. **Anna Bagó‐Mas**: Methodology; validation; formal analysis; investigation; data curation; writing—original draft; writing—review and editing; visualization. **Meritxell Deulofeu**: Investigation; data curation; writing—review and editing. **Judit Homs**: Investigation; writing—review and editing. **Núria Fiol**: Methodology; resources; data curation; writing—review and editing; supervision; project administration; funding acquisition. **Enrique Verdú**: Conceptualization; methodology; resources; data curation; writing—original draft; writing—review and editing; supervision; project administration. **Pere Boadas‐Vaello**: Conceptualization; methodology; formal analysis; resources; data curation; writing—original draft; writing—review and editing; visualization; supervision; project administration; funding acquisition.

## CONFLICT OF INTEREST STATEMENT

The authors declare that the research was conducted in the absence of any commercial or financial relationships that could be construed as being potential conflicts of interest.

### PEER REVIEW

The peer review history for this article is available at https://publons.com/publon/10.1002/brb3.3386.

## Data Availability

The data that support the findings of this study are available from the corresponding author upon reasonable request.
